# Calcium Repletion and Regional Citrate Anticoagulation in Hemodialysis and Hemodiafiltration: Using Dialysate Calcium to Modify Hypocalcemia

**DOI:** 10.1016/j.xkme.2021.05.003

**Published:** 2021-07-15

**Authors:** Justin R. Dorie, Christopher W. McIntyre, Sandrine Lemoine

**Affiliations:** 1 Lilibeth Caberto Kidney Clinical Research Unit, University of Western Ontario, London, Ontario, Canada; 2 Department of Medical Biophysics, University of Western Ontario, London, Ontario, Canada; 3 Lawson Health Research Institute, London, Ontario, Canada; 4 Division of Nephrology, London Health Sciences Centre, London, Ontario, Canada

To the Editor:

The ideal extracorporeal circuit for kidney replacement therapy (CKRT) would have no systemic anticoagulation. However, this approach runs the risk of extracorporeal circuit loss or partial loss of dialyzer fibers. Regional citrate anticoagulation provides anticoagulation of the circuit owing to citrate chelating calcium^1^^,^^2^ but is not usually used with hemodialysis (HD) because of difficulties in implementing it in clinical practice. Regional citrate anticoagulation requires rigorous monitoring of ionized calcium levels,^3^ which leads to increased workload for nurses (frequent modulation of calcium infusion rate is needed to ensure the patient is not exposed to dangerous hypocalcemia) and increases costs significantly related to calcium measurement and replacement infusion supply.

Recent pandemic conditions have posed additional challenges to the use of CKRT in intensive care units. Hypercoagulability, filter loss, nursing workload, and personal protective equipment needs associated with CKRT have all rendered intermittent therapy increasingly preferable. HD is widely considered to be comparable to CKRT.^4^ Accessible HD equipment with easily modifiable dialysate generated from treated tap water requires considerably fewer resources and offers freedom from consumable components. Because regional citrate anticoagulation is not commonly used with HD, we propose the use of a standard HD monitor to provide a hybrid therapy combined with a substantially simplified utilization of regional citrate anticoagulation to address current challenges.

An ex vivo dialysis circuit was set up using donated whole human blood. Ethics committee approval was not required because we used expired donated blood. Dialysis was performed using a Fresenius 5008 machine (Fresenius Medical Care) with online-generated ultrapure dialysate in hemodiafiltration post-dilution mode. A conventional and affordable high-flux synthetic polymer dialyzer (FX 600, Fresenius) was used. Dialysate flow was 500 mL/min, blood pump speed 150 mL/min, and substitutive rate 25 mL/min. Calcium dialysate concentration was increased in increments of 0.25 mmol/L using a standard hemodialysis additive (Baxter Corporation). Anticoagulation was obtained using citrate dextrose solution. Citrate was infused into the heparin infusion port at 200 mL/h and ultrafiltration rate was set at 180 mL/h to account for citrate infusion. Blood samples were obtained pre-dialyzer (n = 4) and post-dialyzer (n = 16) and were analyzed using an i-STAT 1 Analyzer (Abbott Point of Care Inc.). We assessed the following: (1) ionized calcium stability with a fixed calcium dialysate concentration of 1.5 mmol/L. Ionized calcium was tested every hour at 1.5, 2.5, and 3.5 hours. (2) Ionized calcium changes after every 0.25 mmol/L change of calcium dialysate concentration with initial calcium dialysate concentration from 1.25 mmol/L to 2.0 mmol/L (n = 4). Samples were collected every hour (n = 4) and/or 10 minutes after every calcium dialysate concentration change (n = 8).

**Figure 1:**
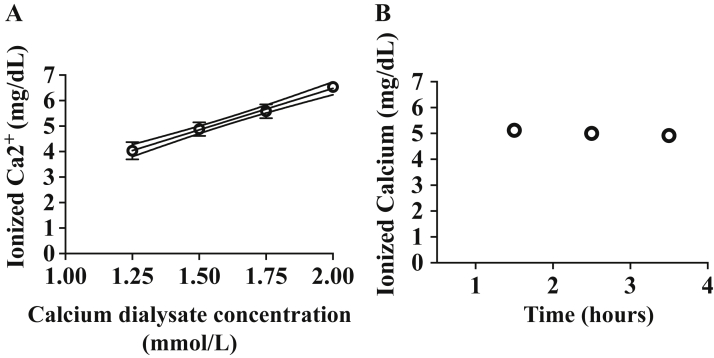
(A) Correlation between mean ± SD ionized calcium levels (mg/dL) and calcium dialysate concentrations (1.25, 1.50, 1.75, and 2.00 mmol/L). (B) Ionized calcium levels (mg/dL) sampled at 1.5, 2.5, and 3.5 hours of simulation using 1.50 mmol/L calcium dialysate concentration in only 1 experiment.

The mean ± standard deviation (SD) pre-dialyzer ionized calcium was 0.26 ± 0.02 mmol/L. The mean ± SD post-dialyzer ionized calcium was 1.01 ± 0.09, 1.22 ± 0.07, 1.39 ± 0.07, and 1.55 ± 0.15 mmol/L for calcium dialysate concentration at 1.25, 1.5, 1.75, and 2 mmol/L, respectively. Ionized calcium was tightly correlated to dialysate calcium concentration, r = 0.99, *P* = 0.001 (Fig 1A). No clotting was observed in any component of the circuit at any time. By using low exchange volume post dilution hemodiafiltration, we were able to further reduce the risk of venous bubble trap clotting by adding additional flow/dilution and vortexing of the blood within the bubble trap, which otherwise could be prone to clotting.

When calcium dialysate concentration was changed by 0.25 mmol/L, the mean ± SD ionized calcium level was 0.20 ± 0.04 mmol/L. When calcium dialysate concentration was modulated, ionized calcium levels were not different when blood was sampled every hour (0.19 ± 0.07 mmol/L, n = 3) compared to every 10 minutes (0.20 ± 0.03 mmol/L, n = 7), *P* = 0.98. For a fixed calcium dialysate concentration of 1.5 mmol/L, ionized calcium was 1.28 mmol/L, 1.25 mmol/L, and 1.23 mmol/L, after 1.5, 2.5, and 3.5 hours, respectively (Fig 1B).

These experiments demonstrate that ionized calcium levels can be managed by modulating calcium dialysate concentrations without the need to administer additional calcium intravenously. We report stable ionized calcium over time to ensure a pre-dialyzer ionized calcium <0.3 mmol/L. We also demonstrated that calcium dialysate concentration changes lead to an immediate modification of ionized calcium levels, since these values were achieved after only 10 minutes, with mass transfer of calcium from dialysate to blood self-regulating as product of the changing relative concentrations within the 2 compartments.

Recent studies have shown patients infected with COVID-19 are severely hypercoagulable,^5^ which can pose a significant risk to maintaining patency of the extracorporeal circuit and may also require a combination of anticoagulants, with heparin-based approaches being inadequate alone.^6^

We present an alternative option to maintain a calcium level target by modulating calcium dialysate concentration, in combination with limited replacement derived from the administration of online-generated dialysate in hemodiafiltration mode, without the need of additional venous calcium infusion. Such an approach is globally available, and is feasible from both a financial and workload perspective. This new protocol could be easily implemented in all HD patients at high risk of bleeding or with heparin contraindications.
